# Leptin–cholesterol crosstalk in pregnancy: Mechanistic implications for placental function and cardiometabolic programming

**DOI:** 10.1113/EP093766

**Published:** 2026-08-11

**Authors:** Julio Flores, Diego Gumera, Andreina Arias, Cristián A. Amador, María José Yañez

**Affiliations:** ^1^ Escuela de Tecnología Médica, Facultad de Ciencias Universidad San Sebastián Santiago Chile

**Keywords:** cardiometabolic disease, cholesterol, hepatic LDL clearance, leptin, leptin resistance, lipoprotein metabolism, PCSK9, placenta, pregnancy

## Abstract

Pregnancy requires the coordinated maternal reorganization of lipid and endocrine pathways to sustain fetal growth and anticipate neonatal nutrition. Leptin, produced by adipose tissue and the placenta, acts as an integrative signal linking maternal energy status with trophoblast function and vascular adaptation, while cholesterol provides essential structural and hormonal substrates required for steroidogenesis and neurodevelopment. Emerging evidence indicates that leptin and cholesterol act as convergent regulators of metabolic and cardiovascular (CV) adaptation during pregnancy. We propose that excessive hyperleptinaemia and maternal hypercholesterolaemia, frequently coexisting, form a convergent axis that modulates placental signalling, alters the epigenetic regulation of lipid transporters and disrupts mitochondrial homeostasis. Mechanistically, this convergence operates through hepatic, epigenetic and renal pathways detailed throughout this review. Beyond the placenta, dysregulation of the leptin–cholesterol axis may extend to maternal organs central to CV homeostasis, particularly the kidney. Emerging evidence suggests that similar leptin‐driven renal pathways may operate during pregnancy, amplifying vascular dysfunction and susceptibility to hypertensive complications. This perspective highlights the leptin–cholesterol axis as a mechanistic link connecting placental, kidney and CV pathways within the Developmental Origins of Health and Disease (DOHaD) paradigm, with potential implications for long‐term cardiometabolic risk. This review integrates molecular, epigenetic and clinical evidence to emphasize lipoprotein‐centred mechanisms, identify critical knowledge gaps and discuss translational opportunities for nutritional intervention, biomarker development and early‐life preventive strategies.

## Abstract (highlights)

- **What is the topic of this review?**
  This review examines the mechanistic interactions between leptin and cholesterol during pregnancy and their convergent roles in placental dysfunction, renal sodium dysregulation and cardiometabolic programming.
- **What advances does it highlight?**
  This review establishes that leptin and cholesterol converge on three mechanistic nodes: leptin upregulates PCSK9, impairs hepatic low density lipoprotein (LDL) clearance and worsens dyslipidaemia; maternal LDL drives epigenetic remodelling at *LEP* and *LRP1* loci, increasing fetal leptin exposure independently of adiposity; and gestational hyperleptinaemia activates renal interleukin‐17A and SGK1 signalling, linking endothelial dysfunction and hypertension. Leptin and cholesterol should be measured together in gestational studies.

## 1 INTRODUCTION

Pregnancy requires the coordinated maternal reorganization of metabolic, immune, endocrine and lipid pathways to sustain fetal growth and prepare for lactation. These physiological adaptations support increased energy demand, steroid hormone synthesis and placental development, while maintaining maternal cardiovascular (CV) homeostasis (Bowman et al., 2021; Niu et al., 2025).

Among the mediators of this adaptation, leptin and cholesterol emerge as central and interconnected regulators. Leptin, secreted by maternal adipose tissue and the placenta, integrates energy availability with trophoblast function, angiogenesis and nutrient transport (Boyle & Le Foll, 2020; Briffa et al., 2015). Cholesterol, in turn, provides an essential substrate for steroidogenesis, membrane biogenesis and fetal neurodevelopment (Lu et al., 2022). Both molecules rise progressively during gestation, reflecting coordinated endocrine and lipid adaptations rather than isolated regulatory processes (Chen et al., 2023; Shen et al., 2022; Yang et al., 2022).

When the increments in leptin and cholesterol levels exceed adaptive thresholds, metabolic and vascular pathology may emerge. Maternal supraphysiological hypercholesterolaemia (MSPH) – defined as total maternal cholesterol concentrations exceeding 280 mg/dL at term (Leiva et al., 2013, 2015) – represents a state in which the physiological gestational rise in lipids becomes maladaptive. In women with MSPH, maternal serum leptin ranged from 15.24 to 42.75 ng/mL (interquartile range 27.52 ng/mL; A. Arias et al., manuscript in preparation) – a distribution that simultaneously spans the first‐trimester threshold associated with incident preeclampsia (≥ 25 ng/mL, odds ratio 18‐fold; Yeboah et al., 2017), the early‐pregnancy threshold predictive of gestational diabetes mellitus (≥ 31 ng/mL, relative risk [RR] 4.7‐fold; Qiu et al., 2004), and the term concentrations reported in pregnancies complicated by intrauterine growth restriction (45–53 ng/mL; Stefaniak & Dmoch‐Gajzlerska, 2022; Yeboah et al., 2017). That a single cohort defined by a cholesterol criterion – without prior selection for obstetric complications – exhibits a leptin distribution spanning all three adverse‐outcome thresholds constitutes direct observational evidence that hyperleptinaemia and MSPH co‐segregate in clinical practice, reinforcing the convergent axis proposed in this review. Leptin and cholesterol dysregulation do not merely co‐occur in complicated pregnancies – they reinforce each other. Leptin upregulates proprotein convertase subtilisin/kexin type 9 (PCSK9) and impairs hepatic low‐density lipoprotein (LDL) clearance (Du et al., 2016; Macchi et al., 2020). In turn, maternal LDL levels modulate DNA methylation at the leptin gene (*LEP*) promoter, altering placental leptin expression (Sletner et al., 2021).

Here, we advance the argument that leptin and cholesterol are not independent mediators of gestational physiology but convergent effectors that reinforce each other's pathological consequences through a shared molecular architecture – centred on the PCSK9–low‐density lipoprotein receptor (LDLR) axis, placental epigenetic remodelling at the *LEP* and *LRP1* loci, and renal tubular sodium (Na^+^) dysregulation. We situate this leptin–cholesterol axis within the Developmental Origins of Health and Disease (DOHaD) paradigm – originally proposed by Barker et al. (1989) and later formalized as a mechanistic framework by Gluckman & Hanson (2004) and Hanson & Gluckman (2014) – a framework proposing that adverse exposures during critical developmental windows permanently alter organ structure and function through epigenetic and metabolic mechanisms, increasing susceptibility to CV, metabolic and kidney disease in adulthood – and argue that understanding this crosstalk has direct implications for how gestational metabolic risk is stratified, monitored and, ultimately, interrupted before its cardiometabolic consequences become irreversible.

## 2 LEPTIN IN REPRODUCTIVE PHYSIOLOGY AND PREGNANCY: PHYSIOLOGICAL ROLES AND MALADAPTIVE EXCESS

Leptin is a 16‐kDa adipocyte‐derived hormone encoded by the *LEP* gene, first identified as a circulating factor responsible for the metabolic and neuroendocrine abnormalities through the *ob*/*ob* mouse model (Zhang et al., 1994). Acting primarily through hypothalamic leptin receptors (Ob‐R), this hormone suppresses appetite, increases energy expenditure and modulates neuroendocrine axes that coordinate metabolism with reproductive and immune function (Campfield et al., 1995; Halaas & Friedman, 1997).

Leptin is also required for follicular growth, oocyte maturation and ovulation (Li et al., 2021); both excessive and insufficient leptin signalling disrupt folliculogenesis and impair oocyte quality (Martins et al., 2021).

Maternal leptin concentrations rise progressively across gestation, reflecting contributions from both adipose depots and the placenta. In particular, the syncytiotrophoblast becomes the dominant endocrine source, accounting for approximately 95% of leptin released into the maternal circulation, with only 5% directed toward the fetal side via villous vascular endothelial cells (Lepercq et al., 2001; Tsai et al., 2015). This trajectory, driven by maternal adiposity and placental synthesis (Hoggard et al., 2001; Rumer et al., 2024), induces a state of central leptin resistance that paradoxically favours increased caloric intake and maternal fat deposition to sustain late gestation demands and the transition to lactation.

In addition to its systemic endocrine role, leptin exerts autocrine and paracrine effects within the placenta itself. It enhances trophoblast proliferation, supports angiogenesis and facilitates amino acid and glucose transport, thereby acting as a local regulator of placental growth and nutrient allocation (Boyle & Le Foll, 2020).

In maternal obesity, augmented circulating leptin arises primarily from expanded adipose tissue and from increased leptin expression in the villous vascular endothelial cells – which preferentially direct their secretion toward the fetal circulation – rather than from enhanced syncytiotrophoblast production, which remains relatively unchanged (Tsai et al., 2015). In pre‐eclampsia (PE), leptin levels are elevated in maternal serum and placental tissue even after accounting for maternal body mass index (BMI), implicating enhanced placental trophoblast production as a primary driver of pathological elevation (Rumer et al., 2024). These source‐specific shifts have differential consequences for maternal and fetal leptin exposure and may explain why the origin of hyperleptinaemia, not merely its absolute concentration, determines downstream pathological outcomes.

Studies in human placenta and maternal circulation consistently demonstrate exaggerated leptin expression in PE, aligning with a vascular phenotype characterized by impaired endothelial homeostasis (Laivuori et al., 2006; Shoemaker et al., 2023; Tsikouras et al., 2025). Importantly, leptin elevations in PE are not uniform across subtypes: serum leptin is significantly higher in early‐onset PE (EOPE, < 34 weeks) than in late‐onset disease (LOPE), and higher in severe than in mild PE, suggesting that leptin concentrations are proportional to disease severity – higher in EOPE and in severe PE – rather than being a non‐specific consequence of hypertension (HTN) (Salimi et al., 2014). This graded relationship raises the possibility that leptin is not simply elevated in PE but is tracking, and potentially amplifying, the pathological process itself. The distinction between EOPE and LOPE reflects disease pathophysiology rather than gestational age per se: while leptin is highest in EOPE – consistent with the greater degree of placental dysfunction characteristic of that phenotype – the predictive association between first‐trimester leptin and subsequent PE has been more robustly demonstrated for term disease (Taylor et al., 2015), possibly because EOPE is a rarer and more heterogeneous phenotype in which placental hypoxia, rather than metabolic excess, may be the dominant upstream driver of hyperleptinaemia. In maternal obesity, placental leptin receptor signalling is itself dysregulated: Janus kinase 2 (JAK2)/signal transducer and activator of transcription 3 (STAT3) and mitogen‐activated protein kinase (MAPK) cascades are perturbed, nuclear factor‐κB (NF‐κB)‐dependent inflammatory programmes are activated and insulin–AKT signalling is impaired (Friis et al., 2013; Misra et al., 2013; Taylor et al., 2015) – a pattern consistent with a state of placental leptin resistance that mirrors the central resistance characteristic of obese non‐pregnant individuals. Concerning the specific end‐organ involvement, the mechanistic evidence linking leptin to vascular endothelial dysfunction is well established: leptin induces oxidative stress (Bouloumie et al., 1999), stimulates endothelin‐1 production in in human endothelial cells (Quehenberger et al., 2002) and promotes suppressor of cytokine signalling 3 (SOCS3)‐mediated JAK2/STAT3 dysregulation in trophoblast cell lines (Hauguel‐de Mouzon & Guerre‐Millo, 2006; Scioscia et al., 2006). In addition, circulating leptin correlates positively with blood pressure in PE, with the strongest associations observed in severe disease (Laivuori et al., 2000; Salimi et al., 2014) – a pattern more consistent with a concentration‐dependent relationship than with leptin as a passive bystander of HTN. Regarding proteinuria, Laivuori et al. (2000) reported a statistically significant but modest positive correlation between serum leptin and urinary protein excretion in preeclamptic women (*r* = 0.305, *P* < 0.05), and experimental infusion of leptin into normotensive pregnant Sprague–Dawley rats produced concurrent and significant increases in systolic blood pressure, 24‐h urinary protein excretion, and serum markers of endothelial activation including E‐selectin and intercellular adhesion molecule‐1 (ICAM‐1) (Ibrahim et al., 2013). These data establish mechanistic plausibility for a leptin–renal injury axis. However, whether leptin drives glomerular injury as an independent effector or whether it operates as an amplifier within a broader inflammatory milieu – placental hypoxia, for instance, simultaneously elevates leptin (Mise et al., 1998) and soluble fms‐like tyrosine kinase‐1 (sFlt‐1) (Faulkner et al., 2022) – remains unknown. This distinction matters for translational purposes: if leptin is a driver, reducing hyperleptinaemia is a therapeutic target; if it is a marker of shared upstream pathology, the target lies upstream.

Experimental evidence positions the renin–angiotensin–aldosterone system (RAAS) as a mechanistic intermediary between leptin and pregnancy‐associated HTN. In obese female mice, exogenous leptin induces HTN and endothelial dysfunction through stimulation of aldosterone synthesis and subsequent endothelial mineralocorticoid receptor (ECMR) activation – an effect abolished by endothelial‐specific ECMR deletion (Huby et al., 2016). More directly relevant to pregnancy, mid‐gestational leptin infusion in mice recapitulates the cardinal features of clinical PE, endothelial dysfunction and fetal growth restriction – through this same leptin–aldosterone–ECMR axis – and this phenotype is similarly abrogated by ECMR deletion (Faulkner et al., 2022). The renal vasculature, including the arterial and afferent/efferent arteriolar endothelium, is among the vascular beds where ECMR is expressed and may therefore contribute to this phenotype, although neither Huby et al. (2016) nor Faulkner et al. (2022) isolated the renal contribution specifically. These findings appear to conflict with a well‐documented clinical observation: in established PE, circulating renin activity and aldosterone concentrations are paradoxically lower than in normotensive pregnancy (Shoemaker et al., 2023). This dissociation, however, does not invalidate the experimental evidence. ECMR is not transcriptionally upregulated by the systemic RAAS; rather, leptin acts upstream to stimulate aldosterone synthesis, which then activates ECMR as a receptor‐level event in target endothelial cells. Because ECMR activation and systemic RAAS output are governed by distinct regulatory mechanisms, elevated ECMR signalling and reduced circulating renin or aldosterone are not mutually exclusive – they can coexist within the same pathological process. Whether this leptin–aldosterone–ECMR pathway operates specifically in the renal vasculature in human PE and contributes to the vascular and renal injury underlying proteinuria and HTN constitutes a clinically important and experimentally testable hypothesis.

Because physiological leptin activity is required for trophoblast proliferation, angiogenesis and placental nutrient transport (Briffa et al., 2015), the therapeutic challenge is how to reduce the pathological excess without compromising its physiological signalling. In human placental explants and trophoblast cell lines, leptin overexposure perturbs canonical JAK2/STAT3 pathways, promotes SOCS3‐mediated feedback inhibition and exacerbates systemic insulin resistance, thereby impairing both metabolic and placental signalling pathways (Hauguel‐de Mouzon & Guerre‐Millo, 2006; Scioscia et al., 2006). This therapeutic constraint underscores that adverse gestational outcomes arise from leptin dysregulation in magnitude or timing, reinforcing the rationale for strategies that target leptin normalization rather than receptor blockade. The connection of follicular development, placental function and gestational HTN suggests that leptin reports metabolic sufficiency to the reproductive system, and when that signal is chronically excessive, each tissue that depends on it for calibration is pushed past its adaptive range (Laivuori et al., 2000).

## 3 CHOLESTEROL'S ROLE IN FEMALE REPRODUCTION AND PREGNANCY: ESSENTIAL SUBSTRATE, INFLUENCING DYSREGULATION AND POSING MATERNAL–FETAL RISK

Maternal cholesterol concentrations progressively increase during gestation, peaking in the third trimester. This maternal physiological hypercholesterolaemia (MPH) supports progesterone and oestrogen biosynthesis, membrane formation and fetal neurodevelopment including myelination and synaptogenesis (Lager & Powell, 2012; Lu et al., 2022), with serum total cholesterol (TC) typically increases 30%–50% and values remaining ≤ 280 mg/dL at term (Barrett et al., 2014).

In almost 30%–44% of pregnancies, maternal cholesterol levels exceed physiological limits, leading to MSPH (Leiva et al., 2013, 2015; Yañez et al., 2026). MSPH is characterized by elevated TC and low‐density lipoprotein (LDL) without corresponding increases in high‐density lipoprotein (HDL) or triglycerides (TG) (Fuenzalida et al., 2020; Leiva et al., 2015), with genetic variability in apolipoprotein E (APOE), LDLR, PCSK9 and scavenger receptor class B type I (SCARB1) implicated as modulators of susceptibility (Cai et al., 2022).

The consequences of MSPH extend beyond maternal dyslipidaemia. Studies have shown links to endothelial dysfunction in both macrovascular and microvascular placental beds (Fuenzalida et al., 2018; Leiva et al., 2013), altered lipid trafficking within trophoblasts (Cai et al., 2020; Fuenzalida et al., 2020; Marseille‐Tremblay et al., 2008; Zhang et al., 2017) and higher cholesterol buildup in fetal arteries. Fetal aortas from MSPH pregnancies often exhibit fatty streaks – the initial histological indicators of atherosclerosis (Napoli et al., 1997, 1999). Longitudinal studies have confirmed that such lesions may persist into childhood and adolescence (de Nigris et al., 2018) and are associated with adverse CV outcomes in adulthood, which include accelerated atherosclerotic lesion progression, dyslipidaemia and increased risk of coronary artery disease (Cacciatore et al., 2022; Mendelson et al., 2016; Muñoz‐Zamorano et al., 2026). The aortic lesions documented in utero (Napoli et al., 1997) and tracked into adolescence (Napoli et al., 1999) are the first histological signs of vascular disease.

In fetal brain tissue, cholesterol is an indispensable structural and signalling substrate – essential for neuronal membrane integrity, synaptogenesis and myelination – and its metabolism is tightly regulated during gestation (Lu et al., 2022). Under normal conditions, excess neuronal cholesterol is eliminated via enzymatic conversion to 24*S*‐hydroxycholesterol (24*S*‐HC) by CYP46A1, which crosses the blood–brain barrier for hepatic clearance (Saeed et al., 2014). However, in animal models of gestational hypercholesterolaemia, disrupted fetal cholesterol homeostasis may overwhelm this clearance pathway, leading to pathological accumulation of 24*S*‐HC and other oxysterols in fetal brain tissue – a shift that, at supraphysiological concentrations, has been associated with pro‐oxidant neuronal toxicity and potentiation of amyloid‐β‐mediated apoptosis in neurons (Björkhem et al., 1998; Gamba et al., 2011; Lu et al., 2022). No study has yet measured 24*S*‐HC directly in fetal brain tissue from MSPH pregnancies. The neurotoxic argument rests on in vitro evidence from neuronal cell lines (Gamba et al., 2011) and on the inference that disrupted fetal cholesterol homeostasis would exceed CYP46A1 clearance capacity – an assumption that requires direct experimental testing in animal models of gestational hypercholesterolaemia. Thus, MSPH not only accelerates vascular pathology in fetal and neonatal vasculature but may also compromise neurological outcomes in the offspring – highlighting cholesterol as a molecule whose essential developmental role is counterbalanced by toxicity when regulatory limits are exceeded.

Beyond total cholesterol concentrations, the qualitative composition of the maternal lipid milieu – particularly the balance between saturated fatty acids and long‐chain polyunsaturated fatty acids (LC‐PUFAs) – exerts independent effects on fetal development. Docosahexaenoic acid (DHA, 22:6*n*–3) and arachidonic acid (ARA, 20:4*n*−6) are selectively transferred across the placenta by membrane fatty acid transport proteins (FATPs), including fatty acid translocase (FAT/CD36), with placental DHA transferred preferentially (Larqué et al., 2011). These LC‐PUFAs are indispensable for fetal brain development and their deficiency during the third trimester ‐when fetal DHA demand increases exponentially‐ has been associated with impaired neurodevelopmental outcomes in the offspring (Coletta et al., 2010; Innis, 2008). Maternal obesity, gestational diabetes and dyslipidaemia alter this selective placental transport, reducing cord blood LC‐PUFA concentrations independently of total lipid levels (Devarshi et al., 2019; Wijendran et al., 2000). Whether hyperleptinaemia specifically modulates placental PUFA trafficking remains an important unresolved question.

## 4 MOLECULAR CROSSTALK BETWEEN LEPTIN AND CHOLESTEROL

**FIGURE 1:**
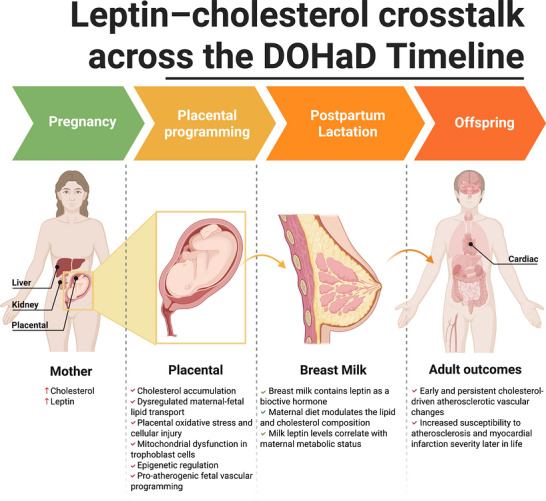
Leptin–cholesterol crosstalk across the DOHaD timeline. During normal pregnancy, coordinated gestational rises in maternal circulating cholesterol and leptin support placental development, steroidogenesis and fetal growth. When these signals exceed physiological thresholds – as occurs in maternal supraphysiological hypercholesterolaemia (MSPH) and hyperleptinaemia – their convergence at the maternal–placental interface initiates a cascade of maladaptive events spanning gestation, lactation and adult offspring health. In the *mother*, gestational dyslipidaemia and hyperleptinaemia activate the hepatic PCSK9–LDLR axis, impairing LDL clearance and amplifying circulating cholesterol excess (Du et al., 2016; Macchi et al., 2020). In the *placenta*, this excess drives cholesterol accumulation in fetal arteries (Napoli et al., 1997, 1999), dysregulated maternal–fetal lipid transport (Fuenzalida et al., 2020; Marseille‐Tremblay et al., 2008), oxidative stress and cellular injury (Fuenzalida et al., 2018, 2020), mitochondrial dysfunction in trophoblast cells (Yañez & Leiva, 2022; Yañez et al., 2026), epigenetic remodelling at the *LEP* and *LRP1* loci that increases fetal leptin exposure independently of maternal adiposity (Guay et al., 2020; Sletner et al., 2021) and pro‐atherogenic fetal vascular changes detectable at delivery (de Nigris et al., 2018; Napoli et al., 1997). During *postpartum lactation*, breast milk leptin – a bioactive hormone that crosses the immature neonatal intestinal barrier – reinforces prenatal metabolic programming (Andreas et al., 2015; Sinkiewicz‐Darol et al., 2022); milk leptin concentrations correlate with maternal metabolic status (Çağiran Yilmaz & Özçelik, 2021; Chatmethakul et al., 2022), and maternal diet further shapes the breast milk lipidome in ways associated with infant growth trajectories (Calvo‐Lerma et al., 2022). In *adult offspring*, these converging prenatal and postnatal exposures manifest as early and persistent cholesterol‐driven atherosclerotic vascular changes (de Nigris et al., 2018; Napoli et al., 1997, 1999) and increased susceptibility to atherosclerosis, dyslipidaemia and greater severity of myocardial infarction in adulthood (Cacciatore et al., 2022; Mendelson et al., 2016; Muñoz‐Zamorano et al., 2026). The mechanistic axes summarized in this figure are detailed in Table 1. DOHaD, Developmental Origins of Health and Disease; LDLR, low‐density lipoprotein receptor; *LEP*, leptin gene; *LRP1*, low‐density lipoprotein receptor‐related protein 1; MSPH, maternal supraphysiological hypercholesterolaemia; PCSK9, proprotein convertase subtilisin/kexin type 9.

The principal molecular axes linking leptin and cholesterol signalling during pregnancy, together with their placental, maternal and long‐term cardiometabolic consequences, are summarized in Table 1. Leptin and cholesterol converge mechanistically at the PCSK9–LDLR axis – documented in HepG2 cells, where leptin upregulates PCSK9 via STAT3 and p38 MAPK, reducing LDLR expression and impairing hepatic LDL clearance (Du et al., 2016; Macchi et al., 2020) (Figure 1). Whether this same convergence operates in the ovarian microenvironment before pregnancy remains poorly described.

**TABLE 1:** Integrated mechanistic pathways linking leptin and cholesterol during pregnancy and their contribution to cardiometabolic programming.

Axis	Core mechanism	Placental/maternal effects	Neonatal/long‐term outcomes	Translational implications	Key references
**Leptin–PCSK9–LDLR**	Leptin upregulates PCSK9 → enhanced LDLR degradation → reduced hepatic LDL clearance	Elevated maternal LDL, placental lipid overload, endothelial dysfunction	Early vascular lipid accumulation, increased cardiometabolic risk	PCSK9 as a biomarker; potential pregnancy‐compatible mechanistic target	Cantin et al. (2022), Du et al. (2016), Macchi et al. (2020), Wang et al. (2022)
**Leptin–ACAT1**	Leptin induces ACAT1 → cholesteryl ester accumulation → foam cell‐like phenotype	Oxidative stress, altered trophoblast lipid handling	Impaired nutrient transfer, fetal metabolic stress	Dietary lipid quality optimization; antioxidant strategies	Hongo et al. (2009)
**Maternal cholesterol– epigenetic and vascular programming**	Maternal LDL fluctuations modulate DNA methylation at *LEP* and *LRP1* loci	Altered placental leptin expression, disrupted lipoprotein transport	Neonatal adiposity, altered hypothalamic programming	Epigenetic biomarkers; maternal lipid monitoring	de Nigris et al. (2018), Guay et al. (2020), Sletner et al. (2021)
**Placental mitochondrial dysfunction**	Excess leptin and cholesterol induce oxidative stress and mitochondrial dysfunction	Reduced placental efficiency, altered energy metabolism	Growth restriction, metabolic vulnerability	Mitochondrial stress markers; antioxidant interventions	Fuenzalida et al. (2020), Yañez & Leiva (2022), Yañez et al. (2026)
**Renal leptin signalling**	Hyperleptinaemia activates renal Ob‐Ra → pro‐inflammatory and profibrotic signalling (TGF‐β1, glomerulosclerosis); and leptin → PCSK9 → IL‐17A → SGK1 → NCC/ENaC → sodium retention → hypertension	Endothelial dysfunction, oxidative stress, reduced leptin clearance	Increased maternal CVD risk, susceptibility to hypertensive disorders	Adipokine ratios as renal/CVD risk markers	Amador et al. (2014), Briffa et al. (2015), Norlander et al. (2016), Wolf et al. (1999)
**Lactation programming**	Altered breast milk leptin and cholesterol reinforce prenatal programming	Postnatal metabolic signal transmission	Childhood adiposity, neurodevelopmental effects	Nutritional counselling during lactation	Andreas et al. (2015), van Brakel et al. (2022)

*Note*: Each axis is described in terms of its core molecular mechanism, downstream placental and maternal effects, neonatal and long‐term offspring outcomes, and potential translational implications. Abbreviations: ACAT1, acyl‐CoA cholesterol acyltransferase 1; CVD, cardiovascular disease; JAK/STAT, Janus kinase/signal transducer and activator of transcription; LDLR, low‐density lipoprotein receptor; *LEP*, leptin gene; *LRP1*, low‐density lipoprotein receptor‐related protein 1; MAPK, mitogen‐activated protein kinase; Ob‐Ra, short isoform of the leptin receptor; PCSK9, proprotein convertase subtilisin/kexin type 9.

Experimental studies have demonstrated that leptin regulates circulating cholesterol levels through the PCSK9–LDLR axis, in which PCSK9, a secreted protein, binds LDLR and targets it for lysosomal degradation, thereby impairing hepatic LDL clearance. In HepG2 cells, leptin up‐regulated PCSK9 gene and protein expression, as well as the phosphorylation of STAT3. Upon STAT3 silencing, leptin lost its ability to activate PCSK9 (Macchi et al., 2020). Specifically, leptin activates the p38 MAPK pathway in hepatocytes, and pharmacological inhibition of p38 MAPK abolishes leptin‐induced upregulation of HNF1α and PCSK9 alongside the concurrent downregulation of LDLR expression and LDL uptake, establishing p38 MAPK as a mechanistic link between leptin signalling and hepatic lipoprotein clearance (Du et al., 2016). PCSK9 transcription in hepatocytes is regulated by two principal mechanisms: sterol‐dependent activation through sterol response element binding proteins‐2 (SREBP‐2) (Jeong et al., 2008) and the hepatocyte nuclear factor 1 alpha (HNF1α) (Li et al., 2009) – the latter being the pathway through which leptin, via p38 MAPK, drives PCSK9 upregulation and concurrent LDLR downregulation (Du et al., 2016). Moreover, leptin upregulates acyl‐CoA cholesterol acyltransferase 1 (ACAT1), driving cholesteryl ester accumulation, foam cell formation and suppression of HDL‐mediated efflux (Hongo et al., 2009). In accordance with this notion, elevated leptin and PCSK9 concentrations have also been correlated with increased endothelial expression of inflammatory mediators, including intercellular adhesion molecule‐1 (ICAM‐1), interferon‐gamma (IFN‐γ) and interleukin (IL)‐17 (Wang et al., 2022). Notably, IL‐17 is not merely a vascular inflammatory mediator – it exerts direct pro‐hypertensive effects through renal mechanisms. Specifically, IL‐17A stimulates serum‐ and glucocorticoid‐regulated kinase 1 (SGK1) in renal tubular cells, maintaining Na^+^‐Cl^−^ cotransporter (NCC) and epithelial Na^+^ channel (ENaC) expression on the luminal surface, thereby enhancing distal tubular Na^+^ and water reabsorption and contributing to blood pressure elevation (Amador et al., 2014; Basile et al., 2021). Amador et al. demonstrated that pharmacological blockade of mineralocorticoid receptor attenuated deoxycorticosterone acetate (DOCA)‐salt‐induced organ damage by suppressing Th17 cell activation and restoring regulatory T lymphocyte populations – establishing a direct mechanistic link between aldosterone‐driven inflammation, IL‐17 signalling and hypertensive end‐organ injury (Amador et al., 2014). Norlander et al. (2016) subsequently identified the specific renal tubular mechanism downstream: IL‐17A maintains NCC and ENaC expression at the luminal surface of distal nephron cells through SGK1‐dependent signalling, with IL‐17A‐deficient mice protected against angiotensin II‐induced Na^+^ transporter upregulation, glomerular injury and blood pressure elevation. Together, these data delineate a coherent molecular sequence in which leptin‐driven upregulation of PCSK9 promotes endothelial expression of ICAM‐1 and IL‐17A, which in turn activates SGK1‐dependent maintenance of NCC and ENaC in the distal nephron, ultimately shifting renal Na^+^ balance toward retention and sustaining blood pressure elevation. We propose that gestational hyperleptinaemia activates this same sequence in the pregnant kidney, providing a mechanistic explanation for why leptin excess in complicated pregnancies is associated with both endothelial dysfunction and Na^+^‐dependent HTN, two features that are rarely dissociated in the clinical setting of PE. This integrated leptin–PCSK9–IL‐17A–renal Na^+^ pathway represents, to our knowledge, a mechanistic connection that has not been explicitly proposed in the obstetric or reproductive physiology literature and is directly testable in existing angiotensin II or DOCA‐salt pregnancy models.

Maternal circulating and placental PCSK9 levels are increased in women with MSPH (Cantin et al., 2022), and plasma leptin independently predicts CV events in non‐pregnant populations (Wolk et al., 2004) – findings that, together with the mechanistic sequence described above, position the leptin–PCSK9–IL‐17A axis as a clinically relevant link between gestational metabolic excess and vascular risk (Figure 1). In this context, PCSK9 inhibition in pregnancy remains pharmacologically unexplored – current guidelines exclude pregnant women from trials of evolocumab and alirocumab, and no reproductive safety data have been reported. Understanding that leptin is an upstream driver of PCSK9 expression in the gestational context reframes the target: controlling hyperleptinaemia through nutritional or metabolic means may achieve an indirect reduction in PCSK9 activity without the teratogenic uncertainty of direct PCSK9 inhibition.

## 5 KIDNEY EFFECTS OF LEPTIN AND DYSLIPIDAEMIA DURING PREGNANCY

Studies in patients have long observed that PE and chronic kidney disease (CKD) share a striking clinical phenotype – HTN, proteinuria and endothelial dysfunction – and that women with pre‐existing CKD face disproportionate gestational risk (Anderson & Ren, 2002; Shankar et al., 2012). This section addresses whether hyperleptinaemia provides the molecular bridge between these two conditions, acting on a kidney already sensitized by the haemodynamic demands of gestation.

Renal function is closely linked to lipid and adipokine homeostasis, and disruptions in either pathway may exacerbate CV and placental risk. In CKD, hyperleptinaemia is a recognized feature, resulting from both reduced renal clearance and increased adipose‐derived leptin production stimulated by hyperinsulinaemia and systemic inflammation (Nordfors et al., 1998; Shankar et al., 2012). Observational studies in humans have linked elevated circulating leptin to renal dysfunction, increased CV risk and greater susceptibility to hypertensive disorders of pregnancy such as PE (Anderson & Ren, 2002; Pérez‐Pérez et al., 2020); however, these associations require cautious interpretation, as hyperleptinaemia and adipose tissue expansion are strongly correlated and difficult to separate as independent contributors in clinical settings. In this context, leptin has been proposed to behave as a molecule with properties akin to uremic toxins, directly inducing proinflammatory and profibrotic effects on renal tissue (Arabi et al., 2022). Importantly, leptin infusion studies in lean and normotensive rats promotes focal glomerulosclerosis, proteinuria, mesangial cell proliferation and oxidative stress (Wolf et al., 2002). In addition, the infusion of leptin into normal rats for 3 weeks induces focal glomerulosclerosis and proteinuria through a paracrine transforming growth factor β (TGF‐β) mechanism: leptin stimulates TGF‐β1 synthesis and type IV collagen production in glomerular endothelial cells, which in turn sensitize mesangial cells via upregulation of TGF‐β type II receptors, promoting extracellular matrix deposition and progressive glomerulosclerosis (Lee et al., 2005; Wolf et al., 1999). This observation is especially relevant during pregnancy, a physiological condition characterized by adaptive hyperleptinaemia, wherein the transition from physiological to pathological leptin exposure remains poorly defined. Whether the magnitude of these renal effects is clinically relevant in the context of gestational hyperleptinaemia – where adiposity, inflammation and haemodynamic changes co‐exist – remains an open question requiring prospective studies in pregnant cohorts.

Consistent with these observations, human cohort studies have indicated that an imbalance of the leptin–adiponectin axis is associated with incident CKD, reinforcing the relevance of adipokine signalling in kidney disease (Zoccali et al., 2002). Notably, a low adiponectin‐to‐leptin ratio has emerged as a strong predictor of CKD development, suggesting that relative leptin excess, rather than absolute adiposity alone, may be particularly detrimental to renal health (Park et al., 2024).

At the molecular level, the kidney exhibits a distinct pattern of leptin receptor expression. In contrast to placenta, where the long signalling‐competent isoform Ob‐Rb predominates, the kidney primarily expresses the short isoform Ob‐Ra in glomerular endothelial cells, mesangial cells and proximal tubular epithelium (Serradeil‐Le Gal et al., 1997). Despite Ob‐Ra's limited JAK–STAT signalling capacity relative to Ob‐Rb, it retains the ability to activate alternative pathways, including PI3K and MAPK, conferring functional responsiveness to circulating leptin (Wolf & Ziyadeh, 2006). The renal consequences of Ob‐Ra activation are context‐ and duration‐dependent: under acute physiological conditions, leptin promotes natriuresis by inhibiting tubular Na^+^ reabsorption through suppression of medullary Na^+^,K^+^‐ATPase activity – an effect that may serve as a short‐term blood pressure buffer (Bełtowski et al., 2002). However, under conditions of chronic hyperleptinaemia, this natriuretic response is blunted and eventually reversed as leptin resistance develops at the tubular level, shifting the renal Na^+^ balance toward retention and contributing to sustained blood pressure elevation (Bełtowski et al., 2010). The implication for complicated pregnancy is direct: if gestational hyperleptinaemia is sustained beyond the acute phase, as it is in PE and obesity, the early natriuretic response is replaced by tubular resistance and Na^+^ retention, contributing to the positive Na^+^ balance that characterizes both conditions. This is not a speculative extrapolation; the temporal shift from natriuresis to Na^+^ retention under chronic leptin exposure has been demonstrated experimentally in rats (Bełtowski et al., 2002, 2010). Activation of Ob‐Ra has additionally been shown to trigger proinflammatory signalling, generate reactive oxygen species and induce profibrotic mediators, including TGF‐β1 and type IV collagen (Wolf et al., 1999) – effects that, in the context of a pregnancy already characterized by systemic endothelial stress, may accelerate the progression from glomerular hyperfiltration to proteinuria and tubular injury.

Sustained leptin signalling through pathways classically associated with central and peripheral leptin action (such as JAK2/STAT3 and MAPK cascades) exacerbates endothelial dysfunction, mesangial expansion and extracellular matrix remodelling in the kidney (Shankar et al., 2012). Beyond glomerular injury, hyperleptinaemia exerts direct haemodynamic effects during pregnancy. In normal pregnant Sprague–Dawley rats, chronic leptin infusion produces a sustained rise in mean arterial pressure of approximately 20 mmHg – a response substantially greater than that observed in non‐pregnant animals – alongside elevated placental tumour necrosis factor‐α and sFlt‐1, key mediators of preeclamptic vascular dysfunction (Palei et al., 2015). However, this effect of leptin is not uniform across experimental models: in rats subjected concurrently to reduced uterine perfusion pressure, leptin infusion did not further elevate blood pressure and was instead associated with a significant reduction in body weight (Palei et al., 2021), and the broader literature reveals comparable heterogeneity depending on dose, timing and the presence of placental ischaemia (Elgazzaz et al., 2025). These findings suggest that hyperleptinaemia may contribute to the hypertensive phenotype of complicated pregnancies in some experimental contexts, but the magnitude and consistency of this effect across models warrant cautious interpretation. In the context of gestational dyslipidaemia, these effects may converge with lipid‐driven vascular stress, reinforcing a shared inflammatory and fibrotic milieu. As renal function declines, leptin clearance falls, circulating concentrations rise further and the kidney is exposed to progressively higher leptin levels – a self‐amplifying dynamic that, in the context of a complicated pregnancy, may accelerate progression toward hypertensive end‐organ damage (Nordfors et al., 1998).

## 6 PLACENTAL MEDIATION AND EPIGENETIC REPROGRAMMING

The evidence that maternal cholesterol directly modifies placental gene methylation – at the LEP promoter (Sletner et al., 2021) and the LRP1 locus (Guay et al., 2020) – establishes that the placenta does not merely respond to the maternal lipid milieu; it is reprogrammed by it, with consequences for fetal leptin exposure and adiposity that are measurable at delivery.

Of the epigenetic mechanisms linking maternal lipid status to fetal outcomes, DNA methylation at specific placental loci is the best characterized. Higher LDL, as observed in MSPH, is associated with reduced methylation, greater placental leptin transcription and higher fetal leptin exposure (Sletner et al., 2021). This is not a modest statistical association: the relationship between maternal LDL and *LEP* methylation persists after adjustment for BMI, gestational age and ethnicity (Sletner et al., 2021), suggesting that the lipid signal reaches the placental epigenome independently of adiposity. Simultaneously, maternal total cholesterol changes across gestation modify methylation at the *LRP1* locus – which encodes the primary placental lipoprotein receptor – and mediation analysis has established a causal pathway from maternal cholesterol fluctuations through *LRP1* methylation to cord blood leptin concentrations (*P* mediation = 0.02) (Guay et al., 2020). Together, these two loci, *LEP* and *LRP1*, position the placental epigenome as an active transducer of maternal lipid signals into fetal adipokine programming, rather than a passive mirror of maternal metabolic status.

In addition, leptin may play a direct role in modulating lipid trafficking across the syncytiotrophoblast. Furthermore, placental leptin may regulate its own fatty acid substrate supply by acting on maternal adipose tissue lipolysis, thereby modulating the availability of non‐esterified fatty acids for placental uptake (Haggarty, 2002). In pregnancies complicated by gestational diabetes or maternal obesity, hyperleptinaemia is associated with altered expression of FATPs, potentially disrupting the selective enrichment of LC‐PUFAs, particularly DHA, in fetal circulation (Haggarty, 2002). Whether excessive leptin signalling specifically impairs DHA transfer to the fetus, analogously to its dysregulation of cholesterol trafficking via the PCSK9–LDLR axis, remains an important unresolved question with direct implications for fetal neurodevelopmental outcomes.

Excessive leptin and cholesterol exposure converge on trophoblast mitochondrial function through overlapping oxidative stress pathways. In MSPH placentas, intracellular cholesterol trafficking is directly disrupted: LDL and HDL uptake by primary human trophoblasts is reduced, SR‐BI expression is decreased and cholesterol efflux patterns are altered – with increased ABCA1 and reduced ABCG1 abundance – suggesting that the trophoblast actively reconfigures its cholesterol handling to limit fetal exposure to maternal cholesterol excess (Fuenzalida et al., 2020; Yañez & Leiva, 2022; Yañez et al., 2026). This reconfiguration altered intracellular sterol distribution impairs lysosomal and mitochondrial function that further compromises trophoblast metabolic efficiency (Yañez & Leiva, 2022; Yañez et al., 2026). Leptin overexposure in trophoblast cell lines dysregulates JAK2/STAT3 signalling and promotes SOCS3‐mediated feedback inhibition (Maymó et al., 2011) – effects that, superimposed on the mitochondrial dysfunction already documented in MSPH trophoblasts (Yañez & Leiva, 2022; Yañez et al., 2026), create a cellular environment in which both signals independently compromise placental metabolic function. Epigenetic data and the functional data together suggest that the placenta is not merely damaged by maternal metabolic excess – it is actively, and imperfectly, trying to compensate for it.

## 7 LACTATION AS A CONTINUATION OF METABOLIC PROGRAMMING

If the intrauterine period sets the metabolic trajectory, lactation is where that trajectory is either reinforced or partially corrected. Breast milk leptin crosses the immature neonatal intestinal barrier and reaches hypothalamic circuits that are still forming (Andreas et al., 2015; Sinkiewicz‐Darol et al., 2022) – meaning that maternal hyperleptinaemia during lactation actively participates in programming the satiety axis of the offspring. Among these, leptin and cholesterol are of particular interest given their established roles in appetite regulation, lipid metabolism and neurodevelopment. Cholesterol, provided in relatively high concentrations during early lactation, acts as an essential structural and metabolic substrate for myelination and synaptogenesis in postnatal brain development (van Brakel et al., 2022).

Leptin levels in breast milk and maternal serum were related to anthropometric measurements of both mothers and infants (Çağiran Yilmaz & Özçelik, 2021). Breast milk from obese or dyslipidaemic mothers exhibits higher leptin and triglyceride levels, and postnatal leptin levels correlate with breast milk leptin content in infants born before 32 weeks of gestation (Chatmethakul et al., 2022), supporting the view that lactation functions as a biological extension of intrauterine metabolic programming.

Excessive leptin exposure during lactation may predispose offspring to early adiposity and altered growth trajectories (Andreas et al., 2015; Vickers et al., 2005), while inadequate cholesterol availability in breast milk has been associated with suboptimal neurodevelopment in neonates and infants (van Brakel et al., 2022). In pregnancies complicated by maternal obesity or gestational diabetes, both leptin and lipid composition of breast milk are altered (Andreas et al., 2015; Panagiotopoulou et al., 2020), with the breast milk lipidome further shaped by maternal diet and associated with infant growth trajectories (Calvo‐Lerma et al., 2022), suggesting that lactation functions as a postnatal extension of the metabolic programming initiated in utero. Whether the lipid and adipokine composition of breast milk from women with MSPH or gestational diabetes differs from that of metabolically healthy mothers, and whether those differences are clinically meaningful for neonatal cardiometabolic programming, is a question the current literature cannot answer. The data on maternal obesity and breast milk composition (Calvo‐Lerma et al., 2022; Sinkiewicz‐Darol et al., 2022) are suggestive but not specific to MSPH. This is a gap that cohort studies with detailed phenotyping of maternal lipid and leptin status during lactation are positioned to address.

## 8 NEONATAL AND LIFELONG CARDIOMETABOLIC IMPLICATIONS

The metabolic consequences of maternal hyperleptinaemia and dyslipidaemia influence neonatal physiology and long‐term cardiometabolic risk. Elevated plasma maternal leptin levels correlate with increased cord blood C‐peptide, an indicator of fetal insulin resistance, suggesting that in utero exposure alters endocrine set points before birth (Misra et al., 2013). Maternal dyslipidaemia similarly contributes to lipid accumulation in fetal arteries and promotes early vascular remodelling, with atherosclerotic lesions detectable in utero (de Nigris et al., 2018; Napoli et al., 1999). The neonatal period is therefore not a clean starting point – it is already a stage in a process that began with the maternal lipid and endocrine environment months earlier.

Evidence from rodent models provides mechanistic support for this programming hypothesis. In rat offspring exposed to a maternal high‐fat diet during gestation, sustained hyperleptinaemia and elevated blood pressure are observed in the absence of postnatal dietary challenge, suggesting epigenetic consolidation of HTN driven by intrauterine lipid overexposure (Lin et al., 2021). In mouse models of gestational hypercholesterolaemia, fetal aortic fatty streak formation is accelerated and persists into postnatal life, with lesion severity correlating with maternal LDL levels (Napoli et al., 1999; Palinski & Napoli, 2002). HTN represents one of the most consistently programmed CV phenotypes in DOHaD animal models, with rodent studies demonstrating that diverse gestational insults – including maternal obesity, protein restriction and glucocorticoid overexposure – converge on elevated offspring blood pressure through shared mechanisms of renal, vascular and neuroendocrine reprogramming (Hsu & Tain, 2021).

Postnatal life offers further opportunities for the reinforcement or mitigation of this programming. In neonates and infants, exposure to altered breast milk composition –characterized by increased leptin and triglycerides in cases of maternal obesity – is associated with early adiposity and suboptimal neurodevelopment in neonates and infants (van Brakel et al., 2022). In childhood and adolescence, longitudinal studies have shown that offspring of mothers with obesity or gestational dyslipidaemia exhibit a higher risk of developing obesity and insulin resistance (Ikenoue et al., 2024; Panagiotopoulou et al., 2020). In adulthood, these trajectories converge toward increased susceptibility to CV disease, consistent with the DOHaD framework (Cacciatore et al., 2022; Mendelson et al., 2016).

Importantly, developmental programming seems to be reversible. In rodent models, neonatal leptin supplementation normalizes adverse metabolic phenotypes – including increased adiposity, hyperphagia and impaired glucose tolerance – induced by intrauterine caloric restriction in rats (Vickers et al., 2005), underscoring a window of postnatal plasticity that may be responsive to early intervention. Whether analogous reprogramming strategies are translatable to human neonates remains an open question; nonetheless, these experimental findings provide a biological rationale for early‐life interventions – nutritional, pharmacological, or lifestyle‐based – aimed at disrupting the intergenerational transmission of cardiometabolic disease.

## 9 DISCUSSION AND CONCLUSION

The central argument of this review is not that leptin and cholesterol are dangerous in pregnancy – they are not at physiological concentrations. The argument is that their co‐elevation in MSPH creates a molecular environment in which each amplifies the pathological effects of the other: leptin‐driven PCSK9 upregulation worsens the dyslipidaemia that characterizes MSPH; maternal LDL excess drives epigenetic changes that increase fetal leptin exposure; and the resulting placental leptin overactivation triggers inflammatory cascades – including IL‐17A‐driven renal Na^+^ retention – that converge on the hypertensive and proteinuric phenotype of complicated pregnancy.

This review integrates insights from molecular signalling pathways, placental epigenetic regulation, renal physiology and clinical outcomes to position leptin–cholesterol crosstalk as a key determinant of maternal–fetal cardiometabolic adaptation within the DOHaD paradigm. The distinction between treating leptin and cholesterol as parallel versus convergent mediators is not semantic. If they are parallel, intervening on one should not be expected to modify outcomes mediated by the other. If they are convergent – operating through shared nodes such as PCSK9, the LEP methylation pathway and the IL‐17A–SGK1 axis – then interventions that reduce hyperleptinaemia may simultaneously attenuate dyslipidaemia, and vice versa. That testable prediction distinguishes this framework from a descriptive co‐occurrence – one that extends existing DOHaD models by incorporating renal and vascular dimensions of the leptin–cholesterol axis. The major molecular axes discussed throughout this review, together with their placental, maternal and offspring‐level consequences, are summarized in Table 1.

From a translational standpoint, the combined assessment of leptin and cholesterol dynamics during pregnancy may improve early risk stratification, enabling the identification of pregnancies and neonates at increased risk for adverse cardiometabolic programming. Maternal nutritional interventions – including omega‐3 fatty acid supplementation and extra virgin olive oil intake – represent biologically plausible and clinically accessible strategies for modulating dyslipidaemia and restoring metabolic balance (Kar et al., 2016; Myatt, 2006). In parallel, the exploitation of early‐life developmental plasticity through targeted neonatal interventions represents a promising, albeit underexplored, opportunity to mitigate maladaptive programming before its establishment as chronic disease.

The mechanistic chain assembled here is traceable from isolated hepatocytes, in which leptin drives PCSK9 upregulation and LDLR degradation (Du et al., 2016; Macchi et al., 2020), through placental tissue at delivery, where maternal LDL has modified LEP and LRP1 methylation (Guay et al., 2020; Sletner et al., 2021), to fetal aortic lesions that persist into adolescence (de Nigris et al., 2018; Napoli et al., 1999). Each link has been documented independently. What this review contributes is their integration into a framework that makes specific predictions: that hyperleptinaemia and MSPH should co‐segregate clinically, that reducing one should attenuate the other through the PCSK9–LDLR axis, and that the renal IL‐17A–SGK1 pathway constitutes a mechanistic explanation for why both hyperleptinaemia and HTN are features of the same pregnancy syndrome.

Several steps in this chain are incompletely characterized. The direct effect of leptin on transplacental DHA flux, the relative contribution of the IL‐17A–renal Na^+^ pathway to clinical PE in humans, and the long‐term cardiometabolic consequences of altered breast milk adipokine composition in MSPH pregnancies all represent tractable experimental questions. What is no longer tractable – or justifiable – is designing gestational metabolic studies that measure leptin or cholesterol in isolation. The evidence reviewed here makes the case that they should always be measured together.

## AUTHOR CONTRIBUTIONS

Conceptualization: Julio Flores, Cristián A. Amador and María José Yañez. Writing‐original draft preparation: Julio Flores, Diego Gumera, Cristián A. Amador and María José Yañez. Writing‐review and editing: Andreina Arias, Cristián A. Amador, and María José Yañez. Funding acquisition: María José Yañez and Cristián A. Amador. All authors have read and approved the final version of this manuscript and agree to be accountable for all aspects of the work in ensuring that questions related to the accuracy or integrity of any part of the work are appropriately investigated and resolved. All persons designated as authors qualify for authorship, and all those who qualify for authorship are listed.

## CONFLICT OF INTEREST

The authors declare no conflicts of interest.

## GENERATIVE AI STATEMENT

No generative AI toolswere used in the preparation of this manuscript.
